# Comparative Untargeted Metabolomic Analysis of Fruiting Bodies from Three *Sanghuangporus* Species

**DOI:** 10.3390/jof11080558

**Published:** 2025-07-28

**Authors:** Zixuan Jiang, Shimao Chen, Jia Song, Tao Xie, Yu Xue, Qingshan Yang

**Affiliations:** 1 College of Pharmacy, Anhui University of Chinese Medicine, Hefei 230012, China; jiangzixuan115@163.com (Z.J.); 15856307880@163.com (S.C.); cici197426@163.com (J.S.); 18715160556@163.com (T.X.); ingxueyu@163.com (Y.X.); 2 Anhui Province Key Laboratory of Research & Development of Chinese Medicine, Hefei 230012, China; 3 Institute of Conservation and Development of Traditional Chinese Medicine Resources, Anhui Academy of Chinese Medicine, Hefei 230012, China

**Keywords:** *Sanghuangporus*, metabolomics, KEGG pathway, species differentiation

## Abstract

*Sanghuangporus* spp. are medicinal fungi with significant therapeutic value, but their taxonomic ambiguity and frequent market adulteration have hindered their standardized utilization. In this study, untargeted metabolomics based on UPLC-Q-TOF-MS was employed to systematically analyze the metabolic profiles of three *Sanghuangporus* species: *Sanghuangporus*. *sanghuang* (SS), *Sanghuangporus*. *vaninii* (SV), and *Sanghuangporus*. *baumii* (SB). A total of 788 metabolites were identified and classified into 16 categories, among which 97 were common differential metabolites, including bioactive compounds such as flavonoids, polysaccharides, and terpenoids. Multivariate statistical analyses (PCA and OPLS-DA) revealed distinct metabolic patterns among the species. KEGG pathway enrichment analysis showed that the differential metabolites were mainly involved in flavonoid and isoflavonoid biosynthesis. Notably, SV and SB exhibited significantly higher levels of several key bioactive compounds, including Apigenin and D-glucuronolactone, compared to SS. These findings highlight substantial interspecies differences in metabolic composition and pharmacological potential, providing a scientific basis for species authentication, quality control, and medicinal development of *Sanghuangporus*.

## 1. Introduction

Sanghuang (*Sanghuangporus* spp.) is a medicinal and edible macrofungus widely distributed across East Asia, particularly in China, Korea, and Japan. Often referred to as “forest gold” [1,2] due to its diverse pharmacological properties, *Sanghuangporus* has been traditionally employed in the prevention and treatment of cancer, diabetes, inflammatory diseases, and circulatory disorders [3,4,5]. These therapeutic effects are primarily linked to its abundant bioactive constituents, notably polysaccharides [6,7], terpenoids [8,9], and flavonoids [1,10], which have demonstrated potent anticancer [11,12], anti-inflammatory [13], antioxidant [14], hypoglycemic [15], and immunomodulatory [16] activities, as well as promoting blood circulation [17]. Such a rich biochemical profile underscores its considerable potential as a valuable resource for developing functional foods and pharmaceuticals.

“Sanghuang” is a general term that refers to a group of morphologically similar fungi. Wu [5] conducted a comprehensive taxonomic revision and proposed that *S. sanghuang* and its closely related species constitute a distinct genus, *Sanghuangporus*. Historically, the identification and classification of *Sanghuangporus* species have relied mainly on morphological traits, leading to taxonomic ambiguities and frequent misidentification in the market. Given that morphologically similar species may differ considerably in their metabolite compositions, comprehensive metabolomic analyses are crucial for accurate species discrimination and effective quality control. Thus, this study employed untargeted metabolomics to systematically investigate the chemical differences among commonly used *Sanghuangporus* species [18]. Notably, given the high market value of *Sanghuangporus. sanghuang* and prevalent adulteration, our metabolomic insights provide a valuable foundation for species authentication and quality assurance.

In recent years, metabolomics has been widely applied to studies on organismal growth, development, and therapeutic interventions [19]. Metabolomics research generally consists of two major approaches: untargeted metabolomics and targeted metabolomics. Untargeted metabolomics is an analytical strategy aimed at identifying as many metabolites as possible in a given sample set [20]. This approach is particularly suitable for characterizing metabolite profiles across different medicinal plant or fungal varieties. When combined with multivariate statistical analysis, it enables the identification of differential metabolites and the characterization of metabolic variation both within and between species [21]. To investigate the chemical differences among commonly used *Sanghuangporus* species in the marketplace, we performed untargeted metabolomic profiling on three representative species: *Sanghuangporus. sanghuang* (SS), *Sanghuangporus. vaninii* (SV), and *Sanghuangporus. baumii* (SB). A series of chemometric analyses were subsequently conducted to identify species-specific metabolites and uncover the distinct metabolic pathways associated with each species.

## 2. Materials and Methods

### 2.1. Sample Information

**Figure 1:**
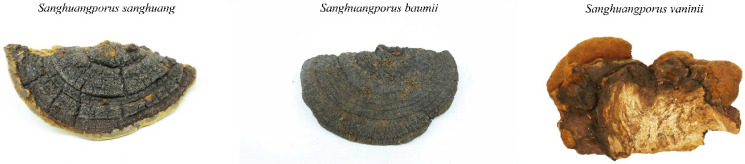
Images of the fruiting bodies of three *Sanghuangporus* species.

In this study, three wild *Sanghuangporus* species were collected in April 2025 from Bomi County in the Nyingchi (Linzhi) region of Tibet, China, at an altitude of approximately 2500 m. Specifically, SS was collected from *Morus alba*, SV from *Populus* spp., and SB from *Syringa* spp. These specimens were taxonomically identified by Associate Professor Qingshan Yang based on detailed morphological characteristics. Each species included six biological replicates, which were stored at −80 °C until metabolomic analysis. The fruiting bodies of these species are shown in Figure 1.

### 2.2. Dry Sample Extraction

Using vacuum freeze-drying technology, the biological samples were placed in a lyophilizer (Scientz-100F, Ningbo Scientz Biotechnology Co., Ltd., Ningbo, China) and then ground (30 Hz, ~1800 rpm, 1.5 min) to powder form by a grinder (MM 400, Retsch GmbH, Haan, Germany). Next, 30 mg of the sample powder was weighed using an electronic balance (MS105DΜ, Mettler Toledo Instruments Co., Ltd., Zurich, Switzerland) and 1500 μL of 70% methanolic aqueous internal standard extract pre-cooled at −20 °C was added (less than 30 mg samples were added with extractant proportionally at 1500 μL per 30 mg). It was vortexed once every 30 min for 30 sec, for a total of 6 times. After centrifugation (rotation speed of 12,000 rpm, 3 min), the supernatant was aspirated, and the sample was filtered through a microporous membrane (0.22 μm pore size) and stored in the injection vial for UPLC-MS/MS analysis.

### 2.3. UPLC-MS/MS Analysis

Samples were analyzed using a UHPLC-MS/MS system (LC-30A coupled with LCMS-8050, Shimadzu, Kyoto, Japan). Chromatographic separation was performed on a Waters ACQUITY UPLC HSS T3 column (1.8 μm, 2.1 × 100 mm) at 40 °C, with a flow rate of 0.4 mL/min and injection volume of 4 μL. The mobile phase consisted of water (0.1% formic acid, A) and acetonitrile (0.1% formic acid, B). The gradient was as follows: 95% A to 35% A in 5 min, then to 1% A in 1 min, held for 1.5 min, returned to initial conditions in 0.1 min, and re-equilibrated for 2.4 min. Mass spectrometry was performed in both positive and negative electrospray ionization modes, operated in information-dependent acquisition (IDA) mode using Analyst TF 1.7.1 software. Nitrogen (N_2_) was used as the sheath, nebulizer, and curtain gas. Source settings: GAS1, 50 psi; GAS2, 60 psi; CUR, 35 psi; temperature, 550 °C; DP, ± 80 V; and ISVF, ± 4500/5500 V. The TOF MS scan range was 50–1250 Da with a 200 ms accumulation. Product ion scans used an accumulation of 40 ms, collision energy of ±30 V, and spread of 15 and monitored up to 12 ions per cycle with a 50 mDa mass tolerance.

### 2.4. Data Processing and Metabolite Identification

Raw mass spectrometry data were first converted to mzML format using ProteoWizard (version 3.0.21252). Peak extraction, alignment, and retention time correction were performed with the XCMS package (version 3.12.0). Peaks with a missing rate greater than 50% across samples were removed. For handling blank values, features with blank ratios exceeding 50% were imputed with one-fifth of the minimum value, while those with blank ratios below 50% were filled using the KNN algorithm combined with one-fifth of the minimum value adjustment. Peak areas were subsequently normalized using the support vector regression (SVR) method.

The filtered peaks were annotated by MS/MS spectral matching against an integrated library comprising our in-house database (MVDB, Metware Database) and publicly available spectral databases such as HMDB (https://hmdb.ca/), KEGG (https://www.kegg.jp/), Mona (https://mona.fiehnlab.ucdavis.edu/), and MassBank (http://www.massbank.jp/). The KEGG Compound and KEGG Pathway databases were additionally employed for metabolite annotation and pathway enrichment analysis. Metabolite identification was performed with a mass accuracy threshold of ≤25 ppm and based on fragmentation pattern similarity. Annotation confidence levels followed the Metabolomics Standards Initiative (MSI) guidelines, primarily corresponding to levels 1–3, indicating putative identification based on spectral similarity or physicochemical properties. Metabolites with a combined identification score above 0.5 and a coefficient of variation (CV) below 0.5 in QC samples were retained. Finally, data from positive and negative ion modes were merged by keeping the identification with the highest qualitative level and lowest CV. The resulting processed data matrix was then used for subsequent statistical analyses.

### 2.5. Data Analysis

Metabolite identification was performed based on an in-house database (MVDB, Metware Database) and publicly available metabolite databases provided by Wuhan MetWare Biotechnology. Following unit variance scaling, unsupervised principal component analysis (PCA) was conducted using the prcomp function in R (version 4.4.3, www.r-project.org). Hierarchical clustering analysis (HCA) of both samples and metabolites is visualized as heatmaps with dendrograms, and Pearson correlation coefficients (PCCs) between samples were calculated using the cor function. Both HCA and PCC visualizations were generated using the ComplexHeatmap R package (version 2.9.4), where normalized metabolite intensities are represented using a continuous color gradient. For pairwise group comparisons, differential metabolites were identified based on variable importance in projection (VIP) scores >1, absolute log2 fold change (|Log2FC|) ≥ 1.0, and FDR-adjusted *p*-values <0.05 to control for multiple testing. VIP scores were derived from OPLS-DA models generated using the MetaboAnalystR package (version 1.0.1), which also provided score plots and permutation tests (200 iterations) to assess model robustness. Prior to OPLS-DA, data were log2-transformed and mean-centered. Metabolite annotation was performed via the KEGG Compound database, and pathway enrichment analysis was conducted using the KEGG Pathway database (http://www.kegg.jp/kegg/pathway.html, accessed on 28 April 2025).

## 3. Result

### 3.1. Total Ion Chromatogram Analysis

**Figure 2:**
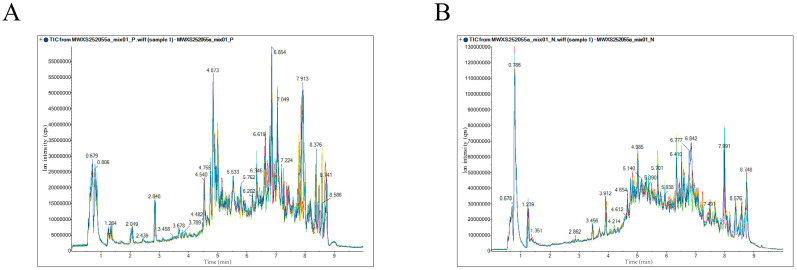
(**A**) The TIC chromatogram of QC samples in positive ion mode; (**B**) the TIC chromatogram of QC samples in negative ion mode. The x-axis represents retention time (minutes), and the y-axis indicates ion intensity expressed in counts per second (cps).

The total ion current (TIC) chromatograms of the quality control (QC) samples under both positive and negative ion modes are shown in Figure 2. The high degree of overlap in peak areas and retention times among all QC samples indicates strong instrument stability and high data quality. Metabolites were identified through spectral matching based on an in-house database (MVDB, Metware Database) and publicly available databases provided by Wuhan MetWare Biotechnology. Specifically, identification criteria included matching accurate mass, retention time, MS/MS fragmentation patterns, and isotopic distribution patterns against authentic reference standards or spectral records. As a result, a total of 435 metabolites were identified in positive ion mode and 353 metabolites in negative ion mode. After removing duplicates identified in both modes, 788 unique metabolites were ultimately confirmed across the three *Sanghuangporus* species.

### 3.2. Classification Statistics of Metabolites in Different Sanghuangporus Species

**Figure 3:**
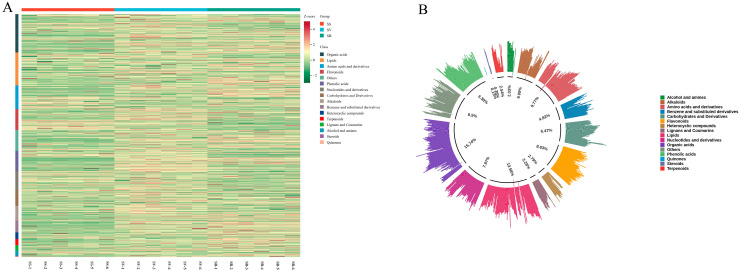
(**A**) A heatmap of all detected metabolites in the three *Sanghuangporus* species; (**B**) a pie chart of metabolite classification in the three *Sanghuangporus* species.

A pie chart of primary metabolite classifications was constructed to visually illustrate the proportional distribution of different chemical categories. As shown in Figure 3, a total of 788 metabolites were identified and classified into the following categories: 124 organic acids, 107 lipids, 77 amino acids and derivatives, 68 flavonoids, 67 compounds categorized as others, 66 phenolic acids, 62 nucleotides and derivatives, 51 carbohydrates and derivatives, 48 alkaloids, 38 benzene and substituted derivatives, 22 heterocyclic compounds, 20 terpenoids, 18 lignans and coumarins, 16 alcohols and amines, 3 steroids, and 1 quinone. The top five categories by proportion were organic acids (15.74%), lipids (13.58%), amino acids and derivatives (9.77%), flavonoids (8.63%), and others (8.50%).

### 3.3. PCA and OPLS-DA

**Figure 4:**
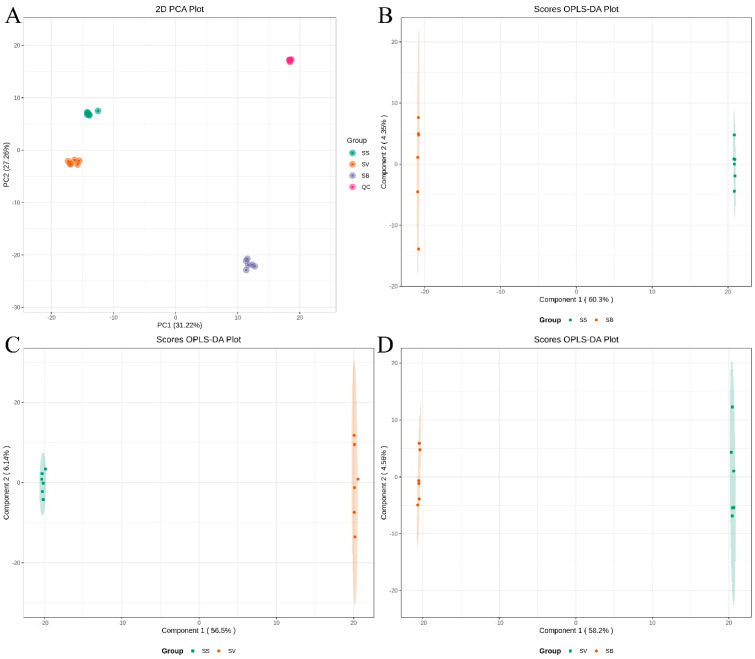
(**A**) A PCA score plot of the three *Sanghuangporus* species and QC samples; (**B**) OPLS-DA of SB vs. SS; (**C**) OPLS-DA of SV vs. SS; (**D**) OPLS-DA of SV vs. SB.

Multivariate statistical analyses, including principal component analysis (PCA) and orthogonal partial least squares discriminant analysis (OPLS-DA), were conducted to reveal the overall metabolic differences among the three *Sanghuangporus* species. PCA is an unsupervised multivariate statistical method commonly used to reduce dimensionality and explore the internal structure of complex datasets by transforming original variables into a smaller number of principal components. In this study, the PCA score plot was used to visualize the metabolic differences among the fruiting bodies of different *Sanghuangporus* species. As shown in Figure 4A, the first principal component (PC1) accounted for 31.22% of the total variance, while the second principal component (PC2) explained 27.26%. The three species formed distinct clusters, indicating clear metabolic differences. In addition, the QC samples were tightly clustered with low variability, demonstrating the high reproducibility and stability of the UPLC-Q-TOF-MS platform. These results confirm that the metabolomic data are robust and suitable for differentiating the fruiting bodies of different *Sanghuangporus* species.

To further confirm and clarify the observed metabolic differences, supervised OPLS-DAs were subsequently performed on pairwise comparisons among the three species (SB vs. SS, SV vs. SS, and SV vs. SB). As presented in Figure 4B–D, the OPLS-DA models effectively enhanced the separation between species groups, underscoring significant metabolic distinctions. Permutation tests (200 iterations) were conducted to evaluate the robustness of these OPLS-DA models, as shown in Figure S1. All models exhibited excellent performance, with high Q^2^ values (>0.9) and R^2^X and R^2^Y values (>0.5), indicating strong predictive and explanatory capabilities. Overall, these complementary multivariate statistical analyses consistently demonstrated clear and significant differences in metabolite composition among the three studied *Sanghuangporus* species, laying a solid statistical foundation for further identification of differential metabolites.

### 3.4. Screening of Differential Metabolites Among the Three Sanghuangporus Species

**Figure 5:**
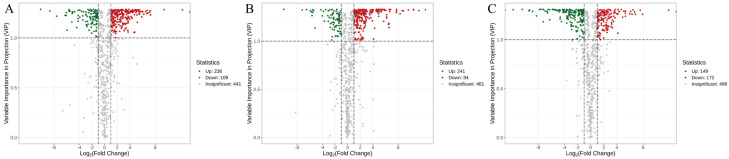
Volcano plots of differential metabolites between the *Sanghuangporus* species: (**A**) SB_vs._SS; (**B**) SV_vs._SS; (**C**) SB_vs._SV. Red dots represent upregulated metabolites, green dots represent downregulated metabolites, and gray dots indicate metabolites with no significant change.

Differential metabolites were screened based on two criteria: an absolute log_2_fold change (|log_2_FC|) ≥1 and a variable importance in projection (VIP) value >1. The results are presented as volcano plots in Figure 5, and selected representative metabolites are listed in Table S1. In the volcano plots, red indicates upregulated metabolites, green indicates downregulated metabolites, and gray indicates metabolites without significant change. Figure 5A shows that a total of 342 differential metabolites were identified between SS and SB, with 236 upregulated and 109 downregulated in SB compared to SS. Figure 5B shows that 321 differential metabolites were found between SS and SV, with 241 upregulated and 84 downregulated in SV compared to SS. Figure 5C reveals 319 differential metabolites between SB and SV, with 149 upregulated and 172 downregulated in SB relative to SV.

To better illustrate the specific differential metabolites and provide biological context, we summarized the top 20 differential abundance metabolites from each pairwise comparison in Table 1. These key metabolites encompass a range of compounds with known or potential pharmacological activities, including phenolic acids, flavonoids, alkaloids, and organic acids. Highlighting these specifically upregulated or downregulated metabolites underscores their role as important differential markers that may drive interspecies metabolic differences. Notably, many of these metabolites—such as naringin, sakuranetin, and cinnamic acid—have been reported to exhibit antioxidant, anti-inflammatory, or antitumor activities, suggesting that their differential accumulation could contribute to the variations in medicinal potential observed among the three *Sanghuangporus* species.

**Figure 6:**
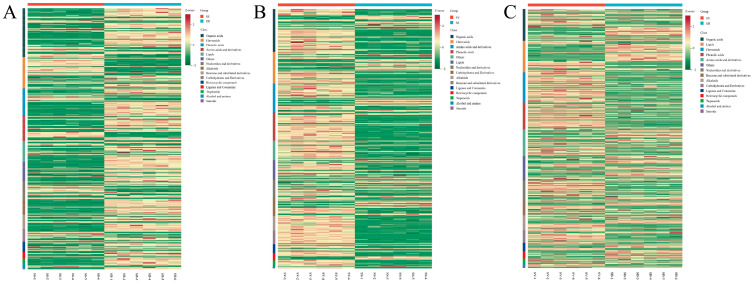
Hierarchical clustering heatmaps of differential metabolites among the *Sanghuangporus* species. (**A**) SB_vs_SS; (**B**) SV_vs_SS; (**C**) SB_vs_SV.

These results indicate that significant differences in the relative abundance of metabolites exist among the different *Sanghuangporus* species. As shown in the heatmaps in Figure 6, both the composition and abundance of metabolites differed markedly among the three species: Figure 6A indicates that SB exhibited significantly higher levels of organic acids, lipids, and terpenoids compared to SS. Figure 6B shows that SV had significantly higher levels of organic acids, amino acids and derivatives, phenolic acids, alkaloids, and benzene and substituted derivatives than SS. Figure 6C demonstrates that SB had higher contents of flavonoids and phenolic acids than SV. These metabolomic differences may underlie interspecies variations in medicinal properties and are consistent with our proposed mechanisms of species-specific bioactivities.

### 3.5. Analysis of Shared Differential Metabolites

**Figure 7:**
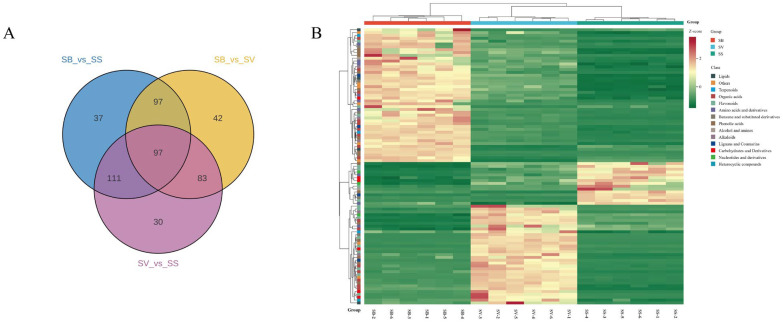
(**A**) A Venn diagram of the differential metabolites in SB vs. SS, SV vs. SS, and SB vs. SV. (**B**) A heatmap of 97 shared differential metabolites among the three groups.

As shown in the Venn diagram in Figure 7A, a total of 97 shared differential metabolites were identified across the three pairwise comparison groups. These metabolites were classified into 14 chemical categories, including 16 organic acids, 16 flavonoids, 15 phenolic acids, 9 amino acids and derivatives, 8 others, 6 carbohydrates and derivatives, 5 alkaloids, 5 benzene and substituted derivatives, 4 lipids, 4 lignans and coumarins, 3 nucleotides and derivatives, 3 terpenoids, 2 alcohols and amines, and 1 heterocyclic compound. Detailed information for these 97 shared differential metabolites is provided in Table S1. As illustrated in Figure 7B, both the number and relative abundance of these 97 shared differential metabolites were markedly higher in SB and SV compared to SS.

### 3.6. Analysis of Flavonoids in the Three Sanghuangporus Species

**Figure 8:**
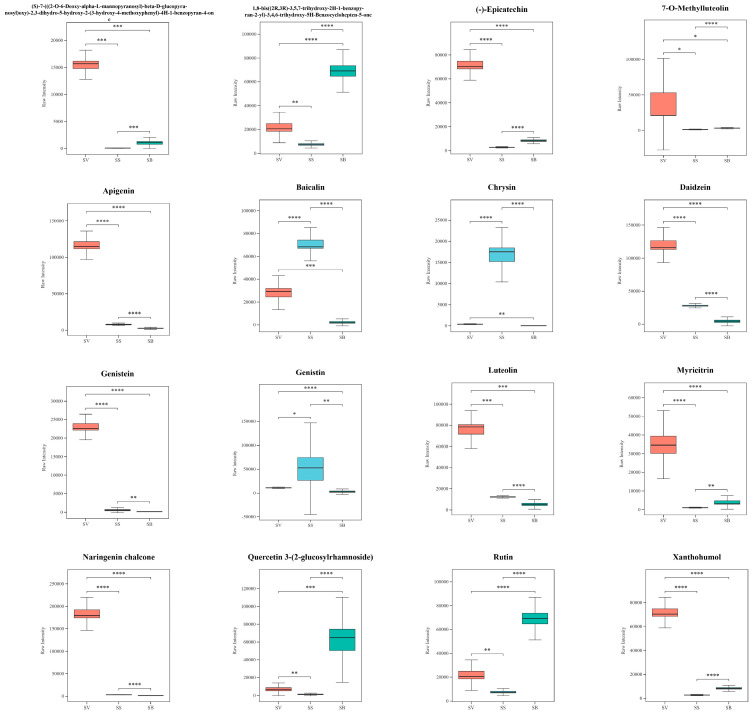
Boxplots of 16 flavonoid metabolites among the 96 shared differential metabolites in the three Sanghuangporus species. N = 6, * *p* < 0.05, ** *p* < 0.02, *** *p* < 0.001, **** *p* < 0.0001.

Numerous studies have demonstrated that flavonoids possess significant biological activities, including antioxidant and antitumor effects [22]. Among the 97 shared differential metabolites identified across the three *Sanghuangporus* species, 16 were flavonoids, whose relative abundances are shown in boxplots (Figure 8). Compounds such as apigenin and (-)-epicatechin have been reported to exert beneficial effects in the prevention and treatment of Alzheimer’s disease [23] and cancer [24,25]. Additionally, flavonoids including luteolin, naringenin chalcone, baicalin, and rutin are known to help reduce the expression of pro-inflammatory cytokines [26,27,28,29], contributing to their anti-inflammatory potential. According to the expression profiles of these 16 flavonoids, 10 metabolites exhibited significantly higher abundance in SS, while 3 flavonoids were more abundant in SV, and another 3 in SB, respectively. Notably, apigenin exhibited approximately a 14.7-fold higher abundance in SV compared to SS, while (-)-epicatechin was approximately 7.0-fold more abundant in SB than in SS.

### 3.7. Analysis of Polysaccharides in the Three Sanghuangporus Species

**Figure 9:**
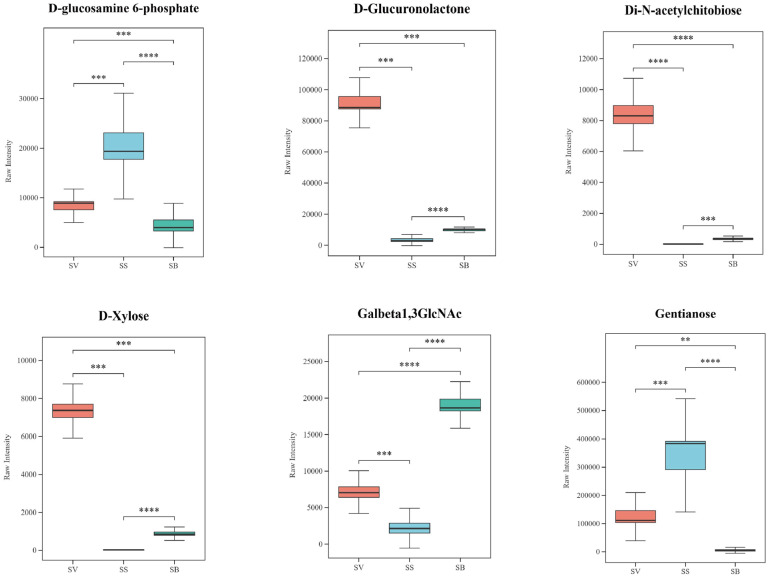
Boxplots of 6 polysaccharide metabolites among the 96 shared differential metabolites in the three *Sanghuangporus* species. N = 6, ** *p* < 0.02, *** *p* < 0.001, **** *p* < 0.0001.

Many fungi in nature contain specific natural bioactive compounds such as polysaccharides, which serve as essential sources of energy and are indispensable to living organisms [30]. In *Sanghuangporus*, polysaccharides are considered the major active constituents and have been shown to exhibit multiple biological functions, including antioxidant, antitumor, immunomodulatory, anti-inflammatory, antiviral, free radical scavenging, and antimicrobial activities. Among the 97 shared differential metabolites, six polysaccharides were identified (Figure 9). Notably, D-glucuronolactone and D-xylose have been reported to possess hepatoprotective and anti-inflammatory properties [31,32]. Both compounds were found at significantly higher levels in SV compared to SS and SB. Specifically, D-glucuronolactone exhibited a 31-fold higher abundance in SV compared to SS, while D-xylose was elevated by 395-fold. Conversely, gentianose was more abundant in SS, showing a 2.6-fold and 66-fold decrease in SV and SB, respectively.

### 3.8. Analysis of Terpenoids in the Three Sanghuangporus Species

**Figure 10:**
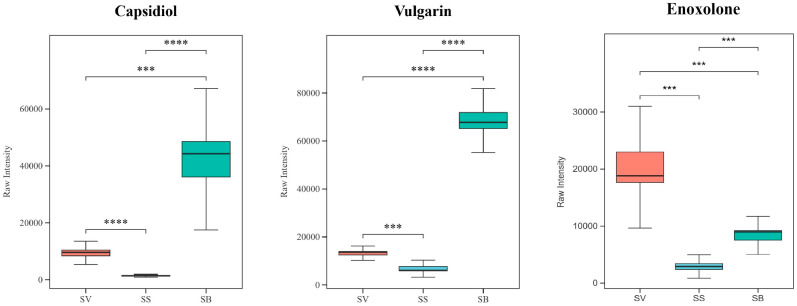
Boxplots of 3 terpenoid metabolites among the 96 shared differential metabolites in the three *Sanghuangporus* species. N = 6, *** *p* < 0.001, **** *p* < 0.0001.

Sesquiterpenoids and triterpenoids in *Sanghuangporus* are considered key pharmacologically active compounds responsible for its medicinal effects, such as antioxidant [33], antibacterial, and antitumor activities [34,35]. In this study, only three terpenoid metabolites were identified as shared differential metabolites, including two sesquiterpenoids (capsidiol and vulgarin) and one triterpenoid (enoxolone). The results (Figure 10) showed that all three terpenoids exhibited lower abundance levels in SS compared to the other two species, SV and SB. Enoxolone exhibited a 6.2-fold higher abundance in SV and a 2.6-fold increase in SB compared to SS. Similarly, capsidiol levels were 6.9-fold higher in SV and 29.5-fold higher in SB, while vulgarin showed a 2.1-fold and 10.8-fold elevation in SV and SB, respectively.

### 3.9. KEGG Pathway Enrichment Analysis of Differential Metabolites

To further elucidate the metabolic pathways involved in the differential metabolites, KEGG pathway enrichment analysis was performed. Metabolite enrichment levels in KEGG pathways often vary between sample groups. In this study, significant enrichment was observed in the flavonoid biosynthesis and isoflavonoid biosynthesis pathways.

**Figure 11:**
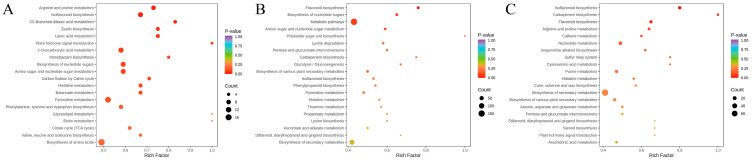
KEGG enrichment bubble diagram of metabolites of different *Sanghuangporus* species. (**A**) SB_vs._SS; (**B**) SV_vs._SS; (**C**) SB_vs._SV.

KEGG pathway enrichment analysis was performed on the differential metabolites identified in the three *Sanghuangporus* species, as shown in Figure 11. In the SV vs. SS group, 198 differential metabolites were mapped to 95 metabolic pathways, with significant enrichment observed in two pathways: flavonoid biosynthesis and biosynthesis of nucleotide sugars. In the SB vs. SS group, 194 differential metabolites were enriched in 95 pathways, with five pathways showing significant enrichment: arginine and proline metabolism, isoflavonoid biosynthesis, C5-branched dibasic acid metabolism, lipoic acid metabolism, and zeatin biosynthesis. In the SB vs. SV group, 189 differential metabolites were associated with 91 pathways, and 3 pathways were significantly enriched: isoflavonoid biosynthesis, carbapenem biosynthesis, and flavonoid biosynthesis.

## 4. Discussion

*Sanghuangporus* is a large wood-inhabiting fungus that has been used in traditional medicine for over 2000 years in China and neighboring countries due to its high medicinal value [36]. To date, 18 species within this genus have been identified, among which 10 are widely distributed across China [37,38]. In this study, metabolomics analysis was employed to systematically investigate the metabolic differences among three representative *Sanghuangporus* species: SS, SV, and SB. The results revealed significant differences in both the composition and abundance of metabolites, particularly in compounds with well-established pharmacological activities, such as flavonoids, polysaccharides, and terpenoids [39]. These findings not only clarify the chemical distinctions among the species but also provide a theoretical basis for understanding their differences in therapeutic efficacy and for promoting the rational utilization of *Sanghuangporus* resources.

In our study, several key metabolites identified across the three *Sanghuangporus* species are closely associated with their traditional medicinal properties. Among these, flavonoids such as naringin [40], sakuranetin [41], and luteolin [42] have been widely reported for their potent antioxidant, anti-inflammatory, and anticancer activities. Polysaccharides including D-xylose [43] are recognized for their immunomodulatory and hepatoprotective effects. Additionally, terpenoids such as capsidiol [44] and vulgarin [45] are known to inhibit tumor growth and modulate oxidative stress. Taken together, the differential accumulation of these metabolites among SS, SV, and SB suggests that they may be major contributors to the variations in pharmacological efficacy observed among these species, thereby underpinning their distinct medicinal values.

In particular, KEGG pathway enrichment analysis revealed significant accumulation of differential metabolites involved in flavonoid and isoflavonoid biosynthesis. Among the isoflavonoid biosynthesis pathway, chlorogenic acid, genistein, and apigenin were notably enriched. These compounds are well-documented for their antioxidant, anti-inflammatory, and estrogen-like activities, which may contribute to immune modulation and cancer prevention. Meanwhile, chrysin, luteolin, and xanthohumol were prominent in the flavonoid biosynthesis pathway and are recognized for their free radical scavenging abilities and roles in inhibiting tumor progression. The differential accumulation of these specific metabolites across SS, SV, and SB further underscores the potential metabolic basis for their distinct therapeutic efficacies. Moreover, these key metabolites were consistently enriched in the flavonoid and isoflavonoid biosynthesis pathways across all three pairwise species comparisons, highlighting their potential role as central contributors to the interspecies variations in pharmacological efficacy, as detailed in Table S2.

While similar classes of metabolites have been widely reported in pharmacological studies of *Sanghuangporus* extracts, most prior work has focused on different extraction methods, cultivation stages, or the mycelia of the same species, often without systematic interspecies comparisons—especially using wild fruiting bodies as performed in this study. This highlights the novelty and significance of our comprehensive metabolic profiling approach. Notably, although these three *Sanghuangporus* species are traditionally used for similar therapeutic purposes, their market prices vary substantially, with SS typically commanding the highest price. However, our data indicate that the less expensive SV and SB tend to exhibit relatively higher levels of key bioactive compounds, suggesting that pharmacological efficacy may not directly correlate with market value. These insights not only advance the biochemical understanding of *Sanghuangporus* but also have practical implications for species authentication, quality control, and the rational utilization and pricing of these valuable medicinal fungi.

Although species within the genus *Sanghuangporus* exhibit close morphological similarities, they can still be reliably distinguished based on comprehensive macroscopic and microscopic characteristics. Therefore, this study employed detailed morphological identification without additional molecular confirmation (e.g., ITS or LSU sequencing). Nonetheless, integrating molecular techniques in future research would further enhance the robustness and accuracy of species authentication.

Admittedly, this study has certain limitations. For instance, the sample sources may vary in environmental conditions, and the age of the fruiting bodies was not strictly controlled, which may affect metabolic outcomes. Additionally, precise geographic coordinates and altitude data were not recorded at the time of collection, potentially limiting the interpretation of specific environmental influences on metabolite composition. Future studies should include larger sample sizes, detailed ecological metadata, and a broader range of *Sanghuangporus* species to improve the generalizability, reproducibility, and environmental contextualization of the findings. In addition, while untargeted metabolomics enables comprehensive detection of metabolites, it may have limited sensitivity for low-abundance compounds or molecules with specific polarity characteristics. Therefore, integrating transcriptomics, proteomics, and other multi-omics approaches may help uncover the regulatory mechanisms underlying the observed metabolic differences. Moreover, in vitro or in vivo functional assays should be incorporated to further elucidate the pharmacological roles of key differential metabolites.

In conclusion, this study provides fundamental data on the metabolic distinctions and potential pharmacological properties of different *Sanghuangporus* species, offering valuable insights for resource utilization, species authentication, and functional research in medicinal fungi.

## 5. Conclusions

In this study, untargeted metabolomics based on UPLC-Q-TOF-MS was employed to systematically analyze the metabolic differences among three commonly used *Sanghuangporus* species: SS, SV, and SB. A total of 788 metabolites were identified, among which 97 differential metabolites were shared across all three species. These differential compounds were primarily associated with biologically active categories such as flavonoids, polysaccharides, terpenoids, phenolic acids, organic acids, and amino acid derivatives. KEGG pathway enrichment analysis revealed significant enrichment in pathways including flavonoid biosynthesis and isoflavonoid biosynthesis. Statistical analyses indicated that SV and SB exhibited a generally higher abundance and diversity of metabolites compared to SS. In particular, key bioactive flavonoids such as apigenin, epicatechin, luteolin, and rutin—known for their antioxidant, anti-inflammatory, and antitumor properties—were more abundant in SV and SB. Moreover, SV was enriched in polysaccharides like D-glucuronolactone and D-xylose, which are associated with hepatoprotective and anti-inflammatory activities. Additionally, terpenoids such as capsidiol and vulgarin, enriched in SB, were also identified as having considerable pharmacological potential. Overall, this study provides a scientific foundation for quality control, species authentication, and resource utilization of the medicinal fungus *Sanghuangporus*. It also lays the groundwork for future investigations into the mechanisms and therapeutic functions of its bioactive components.

## Figures and Tables

**Table 1:** Top 20 differential metabolites (VIP > 1, |Log2FC| ≥ 1, *p* < 0.05) from pairwise comparisons among SB, SV, and SS groups.

Group	Classification	Compound	Up/Downregulated
SB_vs._SS1	Benzene and substituted derivatives	Phenelzine	up
SB_vs._SS2	Organic acids	Geranylgeranyl diphosphate	down
SB_vs._SS3	Phenolic acids	Cinnamic acid	up
SB_vs._SS4	Flavonoids	Naringin	down
SB_vs._SS5	Flavonoids	Chrysin	down
SB_vs._SS6	Alkaloids	Ergothioneine	up
SB_vs._SS7	Organic acids	3-Carboxy-4-methyl-5-propyl-2-furanpropionic acid	up
SB_vs._SS8	Benzene and substituted derivatives	2,3-Dihydroxy-4-methoxybenzoic	up
SB_vs._SS9	Amino acids and derivatives	Glutamylproline	up
SB_vs._SS10	Phenolic acids	4-Hydroxy-3-methoxybenzenemethanol	up
SB_vs._SS11	Benzene and substituted derivatives	Benzimidazole	up
SB_vs._SS12	Amino acids and derivatives	His-Glu	up
SB_vs._SS13	Alcohol and amines	Lipoamide	down
SB_vs._SS14	Organic acids	4-Hydroxycyclohexylcarboxylic acid	up
SB_vs._SS15	Carbohydrates and substituted derivatives	Gentianose	down
SB_vs._SS16	Benzene and substituted derivatives	4-Hydroxytoluene sulfonamide	up
SB_vs._SS17	Flavonoids	Licoisoflavone A	down
SB_vs._SS18	Organic acids	Adenylocuccinic Acid	up
SB_vs._SS19	Phenolic acids	Eudesmic acid	down
SB_vs._SS20	Benzene and substituted derivatives	Cyperine	up
SV_vs._SS1	Others	15-Hydroxynorandrostene-3,17-dione glucuronide	up
SV_vs._SS2	Flavonoids	Sakuranetin	up
SV_vs._SS3	Alkaloids	2-Phenylacetamide	up
SV_vs._SS4	Alkaloids	Dopamine	up
SV_vs._SS5	Organic acids	Geranylgeranyl diphosphate	down
SV_vs._SS6	Phenolic acids	Coniferin	up
SV_vs._SS7	Carbohydrates and derivatives	Di-N-acetylchitobiose	up
SV_vs._SS8	Carbohydrates and derivatives	D-Xylose	up
SV_vs._SS9	Lignans and coumarins	Coumarin	up
SV_vs._SS10	Flavonoids	Naringenin	up
SV_vs._SS11	Flavonoids	(S)-7-(((2-O-6-Deoxy-alpha-L-mannopyranosyl)-beta-D-glucopyranosyl)oxy)-2,3-dihydro-5-hydroxy-2-(3-hydroxy-4-methoxyphenyl)-4H-1-benzopyran-4-one	up
SV_vs._SS12	Lignans and coumarins	Matairesinol	up
SV_vs._SS13	Flavonoids	Naringin	down
SV_vs._SS14	Others	Resveratrol	up
SV_vs._SS15	Organic acids	Porphobilinogen	up
SV_vs._SS16	Phenolic acids	Salicin	up
SV_vs._SS17	Alkaloids	Pyridoxamine	up
SV_vs._SS18	Phenolic acids	Agnuside	up
SV_vs._SS19	Others	12a-Hydroxyrotenone	up
SV_vs._SS20	Flavonoids	alpha-Mangostin	down
SB_vs._SV1	Benzene and substituted derivatives	Phenelzine	up
SB_vs._SV2	Flavonoids	Sakuranetin	down
SB_vs._SV3	Alkaloids	Dopamine	down
SB_vs._SV4	Phenolic acids	1′-Acetoxychavicol acetate	down
SB_vs._SV5	Others	2-Aminoacetophenone	down
SB_vs._SV6	Lignans and coumarins	Coumarin	down
SB_vs._SV7	Lipids	3-Hydroxyhexadecanoic acid	down
SB_vs._SV8	Benzene and substituted derivatives	Benzimidazole	up
SB_vs._SV9	Phenolic acids	Agnuside	down
SB_vs._SV10	Flavonoids	Naringenin	down
SB_vs._SV11	Phenolic acids	Cinnamic acid	up
SB_vs._SV12	Others	15-Hydroxynorandrostene-3,17-dione glucuronide	down
SB_vs._SV13	Flavonoids	Genistein	down
SB_vs._SV14	Flavonoids	Naringenin chalcone	down
SB_vs._SV15	Phenolic acids	Coniferin	down
SB_vs._SV16	Phenolic acids	4-Hydroxy-3-methoxycinnamaldehyde	up
SB_vs._SV17	Lipids	17,18-EpETE	up
SB_vs._SV18	Organic acids	Porphobilinogen	down
SB_vs._SV19	Alkaloids	Pyridoxamine	down
SB_vs._SV20	Flavonoids	5,7-Dihydroxy-2-(4-hydroxy-3-methoxy-phenyl)-3,6,8-trimethoxy-chromen-4-one	down

## Data Availability

The raw data supporting the conclusions of this article will be made available by the authors on request.

## Supplementary Materials

The following supporting information can be downloaded at: https://www.mdpi.com/article/10.3390/jof11080558/s1, Figure S1: Permutation tests for three group comparisons: Table S1: A total of 97 common differential metabolites among SS vs. SB, SS vs. SV, and SV vs. SB. Table S2: Differential metabolites mapped to the enriched flavonoid and isoflavonoid biosynthesis pathways across all three pairwise comparisons among SS vs. SB, SS vs. SV, and SV vs. SB.

## References

[1] Zhou Z., Liang S., Zou X., Teng Y., Wang W., Fu L. (2023). Determination of Phenolic Acids Using Ultra-High-Performance Liquid Chromatography Coupled with Triple Quadrupole (UHPLC-QqQ) in Fruiting Bodies of *Sanghuangporus baumii* (Pilát) LW Zhou and YC Dai. Plants.

[2] Luo Y., Cao N., Huang L., Tang L., Liu X., Zhang W., Huang S., Xie X., Yan Y. (2024). Structural Characterization, and Antioxidant, Hypoglycemic and Immunomodulatory Activity of Exopolysaccharide from Sanghuangporus sanghuang JM-1. Molecules.

[3] Lu J., Su M., Zhou X., Li D., Niu X., Wang Y. (2024). Research Progress of Bioactive Components in Sanghuangporus spp. *Molecules*
**2024**, *29*,1195. Molecules.

[4] Zhang X., An S., Zhou L., Bao Y. (2025). Comparison of Morphology, Components, and Activity of Four Species of *Sanghuangporus Mushrooms* (Agaricomycetes). Int. J. Med. Mushrooms.

[5] Wu S.-H., Dai Y.-C., Hattori T., Yu T.-W., Wang D.-M., Parmasto E., Chang H.-Y., Shih S.-Y. (2012). Species clarification for the medicinally valuable’sanghuang’mushroom. Bot. Stud..

[6] Cheng J., Song J., Wei H., Wang Y., Huang X., Liu Y., Lu N., He L., Lv G., Ding H. (2020). Structural characterization and hypoglycemic activity of an intracellular polysaccharide from *Sanghuangporus sanghuang* mycelia. Int. J. Biol. Macromol..

[7] Sułkowska-Ziaja K., Balik M., Muszyńska B. (2021). Selected Species of the Genus Phellinus-Chemical Composition, Biological Activity, and Medicinal Applications. Chem. Biodivers..

[8] Liu Z., Liu R., Tong X., Zou L. (2022). New Insights into Methyl Jasmonate Regulation of Triterpenoid Biosynthesis in Medicinal Fungal Species *Sanghuang porusbaumii* (Pilát) LW Zhou & YC Dai. J. Fungi..

[9] Wang X., Sun J., Wang S., Sun T., Zou L. (2023). Salicylic acid promotes terpenoid synthesis in the fungi *Sanghuangporus baumii*. Microb. Biotechnol..

[10] Wang S., Liu Z., Wang X., Liu R., Zou L. (2022). Mushrooms Do Produce Flavonoids: Metabolite Profiling and Transcriptome Analysis of Flavonoid Synthesis in the Medicinal Mushroom *Sanghuangporus baumii*. J. Fungi.

[11] He P.Y., Hou Y.H., Yang Y., Li N. (2021). The anticancer effect of extract of medicinal mushroom *Sanghuangprous vaninii* against human cervical cancer cell via endoplasmic reticulum stress-mitochondrial apoptotic pathway. J. Ethnopharmacol..

[12] Wan X., Jin X., Xie M., Liu J., Gontcharov A.A., Wang H., Lv R., Liu D., Wang Q., Li Y. (2020). Characterization of a polysaccharide from *Sanghuangporus vaninii* and its antitumor regulation via activation of the p53 signaling pathway in breast cancer MCF-7 cells. Int. J. Biol. Macromol..

[13] Zuo K., Tang K., Liang Y., Xu Y., Sheng K., Kong X., Wang J., Zhu F., Zha X., Wang Y. (2021). Purification and antioxidant and anti-Inflammatory activity of extracellular polysaccharopeptide from sanghuang mushroom, *Sanghuangporus lonicericola*. J. Sci. Food Agric..

[14] Cai C., Ma J., Han C., Jin Y., Zhao G., He X. (2019). Extraction and antioxidant activity of total triterpenoids in the mycelium of a medicinal fungus, *Sanghuangporus sanghuang*. Sci. Rep..

[15] Rajput S.A., Wang X.Q., Yan H.C. (2021). Morin hydrate: A comprehensive review on novel natural dietary bioactive compound with versatile biological and pharmacological potential. Biomed. Pharmacother..

[16] Jung G.H., Kang J.H. (2020). Efficacy of *Phellinus linteus* (sanghuang) extract for improving immune functions: Study protocol for a randomized, double-blinded, placebo-controlled pilot trial. Medicine.

[17] Wang H., Ma J.X., Zhou M., Si J., Cui B.K. (2022). Current advances and potential trends of the polysaccharides derived from medicinal mushrooms sanghuang. Front. Microbiol..

[18] Wu S.H., Chang C.C., Wei C.L., Jiang G.Z., Cui B.K. (2019). *Sanghuangporus toxicodendri* sp. nov. (Hymenochaetales, Basidiomycota) from China. MycoKeys.

[19] Gika H., Virgiliou C., Theodoridis G., Plumb R.S., Wilson I.D. (2019). Untargeted LC/MS-based metabolic phenotyping (metabonomics/metabolomics): The state of the art. J. Chromatogr. B.

[20] Zhao X., Chen M., Zhao Y., Zha L., Yang H., Wu Y. (2019). GC–MS-Based Nontargeted and Targeted Metabolic Profiling Identifies Changes in the Lentinula edodes Mycelial Metabolome under High-Temperature Stress. Int. J. Mol. Sci..

[21] Bobiş O., Bonta V., Cornea-Cipcigan M., Nayik G.A., Dezmirean D.S. (2021). Bioactive Molecules for Discriminating Robinia and Helianthus Honey: High-Performance Liquid Chromatography-Electron Spray Ionization-Mass Spectrometry Polyphenolic Profile and Physicochemical Determinations. Molecules.

[22] Wang Y., Zhang C., Zhao Y., Wu F., Yue Y., Zhang Y., Li D. (2025). Ultrasound-assisted optimization extraction and biological activities analysis of flavonoids from Sanghuangporus sanghuang. Ultrason. Sonochem..

[23] Zhang N., Nao J., Dong X. (2025). Efficacy and Safety of Natural Apigenin Treatment for Alzheimer′s Disease: Focus on In vivo Research Advancements. Curr. Neuropharmacol..

[24] Peng C., Zhang X., Zhou N., Hu T., Shen Y., Chen T.J., Liu Y., Cui H., Zhu S. (2024). Apigenin inhibits lipid metabolism of hepatocellular carcinoma cells by targeting the histone demethylase KDM1A. Phytomedicine.

[25] Pereyra-Vergara F., Olivares-Corichi I.M., Luna-Arias J.P., Méndez-Luna D., García-Sánchez J.R. (2025). Epicatechin Decreases UCP2 Gene Expression in MDA-MB-231 Breast Cancer Cells by the Presence of a Regulatory Element in the Promoter. Int. J. Mol. Sci..

[26] Hirai S., Kim Y.I., Goto T., Kang M.S., Yoshimura M., Obata A., Yu R., Kawada T. (2007). Inhibitory effect of naringenin chalcone on inflammatory changes in the interaction between adipocytes and macrophages. Life Sci..

[27] Guo L.T., Wang S.Q., Su J., Xu L.X., Ji Z.Y., Zhang R.Y., Zhao Q.W., Ma Z.Q., Deng X.Y., Ma S.P. (2019). Baicalin ameliorates neuroinflammation-induced depressive-like behavior through inhibition of toll-like receptor 4 expression via the PI3K/AKT/FoxO1 pathway. J. Neuroinflammation.

[28] Deng L., Yu Q., Kuang G., Wang L., Fan J., Ye L. (2025). Luteolin modulates liver macrophage subtype polarization and play protective role in sepsis induced acute hepatic injury. Inflamm. Res..

[29] Wang X., Gong M., Zhu Z., Zhang B., Han L., Li W., Wu Z., Ma Q., Wang Z., Qian W. (2025). Rutin protects the pancreas from inflammatory injury and oncogene-driven tumorigenesis by inhibiting acinar to ductal metaplasia. Eur. J. Pharmacol..

[30] Wang Y., Song X., Wang Z., Li Z., Geng Y. (2023). Effects of Pine Pollen Polysaccharides and Sulfated Polysaccharides on Ulcerative Colitis and Gut Flora in Mice. Polymers.

[31] Liu G., Das S.K. (2025). D-Xylose Ameliorates Non-Alcoholic Fatty Liver Disease by Targeting Macrophage-expressed LYZ Gene. Cell Biochem. Biophys..

[32] Shen Y., Miao Z., Zheng Y., Dong Y., Han M., Huang C., Bai R., Xia C., Shi S., Li J. (2025). D-Glucuronolactone Supplementation Enhances Production Performance, Eggshell Quality, and Liver Health in Laying Hens. Animals.

[33] Khadbaatar S., Bao H., Gao X., Huo H. (2024). Study on Differences of Metabolites among Different Ganoderma Species with Comprehensive Metabolomics. J. Fungi..

[34] Zhang D.X., Liu B.Y., Xue F.F., Tang Y.L., Yan M.J., Wang S.X., Guo L., Tong T., Wan L.N., Liu Y.N. (2025). Paclobutrazol induces triterpenoid biosynthesis via downregulation of the negative transcriptional regulator SlMYB in Sanghuangporus lonicericola. Commun. Biol..

[35] Tian C., Sun L.T., Jin T., Yuan L.L., Xu W.F., Yang H.X., Feng T., Liu J.K. (2024). Phellintremulins A-C, antinociceptive sesquiterpenoids from the medicinal fungus Phellinus tremulae. Phytochemistry.

[36] Lin W.C., Deng J.S., Huang S.S., Wu S.H., Chen C.C., Lin W.R., Lin H.Y., Huang G.J. (2017). Anti-Inflammatory Activity of *Sanghuangporus sanghuang* Mycelium. Int. J. Mol. Sci..

[37] Chen J.H., Shen S., Zhou L.W. (2022). Modeling current geographic distribution and future range shifts of Sanghuangporus under multiple climate change scenarios in China. Front. Microbiol..

[38] Shen S., Liu S.L., Jiang J.H., Zhou L.W. (2021). Addressing widespread misidentifications of traditional medicinal mushrooms in *Sanghuangporus (Basidiomycota)* through ITS barcoding and designation of reference sequences. IMA Fungus.

[39] Qi Y., Guo X.Y., Xu X.Y., Hou J.X., Liu S.L., Guo H.B., Xu A.G., Yang R.H., Yu X.D. (2024). Widely targeted metabolomics analysis of *Sanghuangporus vaninii* mycelia and fruiting bodies at different harvest stages. Front. Microbiol..

[40] Alhalmi A., Amin S., Ralli T., Ali K.S., Kohli K. (2025). Therapeutic role of naringin in cancer: Molecular pathways, synergy with other agents, and nanocarrier innovations. Naunyn Schmiedebergs Arch. Pharmacol..

[41] Alharbi K.S., Alenezi S.K., Alsahli T., Afzal M., Mantargi M.J.S., Kazmi I., Sayyed N. (2025). Effect of sakuranetin against cyclophosphamide-induced immunodeficiency mice: Role of IFN-γ/TNF-α/IgG/IgM/interleukins. Naunyn. Schmiedebergs Arch. Pharmacol..

[42] Jiang Z.B., Wang W.J., Xu C., Xie Y.J., Wang X.R., Zhang Y.Z., Huang J.M., Huang M., Xie C., Liu P. (2021). Luteolin and its derivative apigenin suppress the inducible PD-L1 expression to improve anti-tumor immunity in KRAS-mutant lung cancer. Cancer Lett..

[43] Song G., Wang K., Zhang H., Sun H., Wu B., Ju X. (2017). Structural characterization and immunomodulatory activity of a novel polysaccharide from Pteridium aquilinum. Int. J. Biol. Macromol..

[44] Pierman B., Toussaint F., Bertin A., Lévy D., Smargiasso N., De Pauw E., Boutry M. (2017). Activity of the purified plant ABC transporter NtPDR1 is stimulated by diterpenes and sesquiterpenes involved in constitutive and induced defenses. J. Biol. Chem..

[45] Zhang L., Liu X., Xu M., Cheng X., Li N., Xu H., Feng Y., Guan T., Xiao L. (2025). Patrinia scabiosaefolia L. Modulates the Intestinal Microecology to Treat DSS-Induced Ulcerative Colitis: UHPLC-OE-MS/MS, Network Pharmacology, and Experimental Validation. Foods.

