# Supplementary material for: Comparative Untargeted Metabolomic Analysis of Fruiting Bodies from Three Sanghuangporus Species
Source: J Fungi (Basel). 2025 Jul 28;11(8):558. doi: 10.3390/jof11080558 (PMC12387423; doi:10.3390/jof11080558)
Supplement: Supplementary file 1 [file jof-11-00558-s001.zip › Figure S1.pdf]

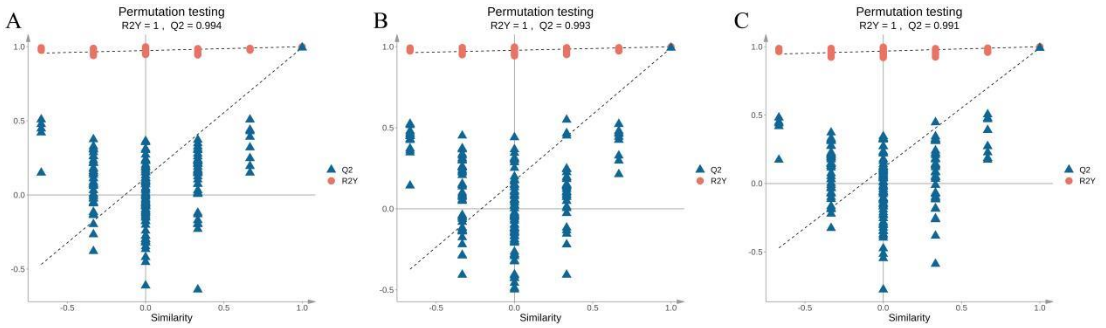

Figure S1. Permutation tests for the OPLS-DA models of (A) SB vs SS, (B) SB vs. SV, and (C) SV vs. SS.Red circles represent R^2Y values and blue triangles represent Q^2 values. The Q^2 intercepts indicate that the models are robust and not overfitted.
